# Methicillin-resistant *Staphylococcus aureus* (MRSA) in East Africa: red alert or red herring?

**DOI:** 10.1186/s12879-019-4245-3

**Published:** 2019-07-09

**Authors:** Frederick K. Wangai, Moses M. Masika, Marybeth C. Maritim, R. Andrew Seaton

**Affiliations:** 1 Unit of Clinical Infectious Diseases, Department of Clinical Medicine and Therapeutics, School of Medicine, College of Health Sciences-University of Nairobi, P.O. Box 19676, Nairobi, 00202 Kenya; 2 Department of Microbiology, School of Medicine, College of Health Sciences-University of Nairobi, P.O. Box 19676, Nairobi, 00202 Kenya; 3 Consultant in Infectious Diseases and General Medicine, Antimicrobial Management Team Lead NHS Greater Glasgow and Clyde Health Board, Queen Elizabeth University Hospital, 1345 Govan Road, Glasgow, G51 4TF United Kingdom

**Keywords:** Methicillin-resistant *Staphylococcus aureus*, Antimicrobial resistance, VITEK, Methicillin, Cefoxitin

## Abstract

**Background:**

Methicillin-resistant *Staphylococcus aureus* (MRSA) is associated with significant morbidity and mortality and has resultant important economic and societal costs underscoring the need for accurate surveillance. In recent years, prevalence rates reported in East Africa have been inconsistent, sparking controversy and raising concern.

**Methods:**

We described antimicrobial susceptibility patterns of *Staphylococcus aureus* isolates cultured from patients within the Internal Medicine department of the largest public healthcare facility in East and Central Africa- the Kenyatta National Hospital (KNH) in Nairobi, Kenya. Routine antimicrobial susceptibility data from non-duplicate *Staphylococcus aureus* isolates cultured between the years 2014–2016 from the medical wards in KNH were reviewed.

**Results:**

Antimicrobial susceptibility data from a total of 187 *Staphylococcus aureus* isolates revealed an overall MRSA prevalence of 53.4%. Isolates remained highly susceptible to linezolid, tigecycline, teicoplanin and vancomycin.

**Conclusions:**

The prevalence of MRSA was found to be much higher than that reported in private tertiary facilities in the same region. Careful interrogation of antimicrobial susceptibility results is important to uproot any red herrings and reserve genuine cause for alarm, as this has a critical bearing on health and economic outcomes for a population.

**Electronic supplementary material:**

The online version of this article (10.1186/s12879-019-4245-3) contains supplementary material, which is available to authorized users.

## Background

*Staphylococcus aureus* has generated a lot of interest over the last half century due to its ability to rapidly adapt to antibiotic pressure and develop antibiotic resistance [1]. The health burden attributable to Methicillin-resistant *Staphylococcus aureus* (MRSA) has been summarised in the World Health Organization Antimicrobial Resistance report as significant increased all-cause, bacterium-attributable and intensive care unit (ICU) mortality; as well as post-infection and ICU length of stay. MRSA species has been shown to demonstrate higher rates of associated septic shock and discharge to long-term care than methicillin-susceptible species [2]. The economic impact of MRSA as measured through resource-use outcomes showed extended duration of hospital and ICU length of stay, as well as greater proportion of discharges to long-term healthcare facilities. Overall, this implies higher resource utilisation in treatment of MRSA infections both in the acute setting and long term. Increased burden on healthcare resources attributable to MRSA is widely known globally as it has been reported to account for more than 60% of *Staphylococcus aureus* isolates causing nosocomial infection in intensive care units (ICUs) in the United States [3–6].

Reports of Methicillin resistance in *Staphylococcus aureus* is documented to have exceeded 20% in all World Health Organization (WHO) regions, and above 80% in some regions [2]. In Africa, MRSA prevalence intra-country and inter-country has been reported to be heterogenous [7]. National data from 9 African countries shows MRSA rates to approximate between 12 and 80%, with some countries exceeding 82% [6, 8]. For example, in East Africa, high prevalence rates of between 31.5 to 42% among patients and healthcare workers have been recorded in Uganda [9, 10], 31 to 82% MRSA prevalence in Rwanda [11, 12], and in 10 to 50% in Tanzanian studies [13–21]. However, there have been some pockets of positive reports owing to antimicrobial stewardship and infection control practices such as South Africa, which recorded a modest decline from 34 to 28% since 2011 [6, 8, 22].

Antimicrobial resistance (AMR) data from Kenya has been variable and inconsistent, due to lack of effective and systematic routine surveillance systems [22, 23]. Due to its contribution to health and economic outcomes on a global scale, there is a need for accurate up-to-date data on MRSA resistance and its surveillance. Inconsistent prevalence rates, highly variable or even erroneous AMR estimates presenting a false alarm may unnecessarily startle policy makers and healthcare facility administrators into action [24]. This inadvertently bears economic implications in view of the extra resources used in laboratory methods, clinical processes, inaccurate interventions and poor investment decisions [25]. Ultimately, such disparities in local and regional resistance data make it difficult to extrapolate relevant categorical conclusions [8].

## Methods

### Study setting

The Kenyatta National Hospital (KNH) is the largest teaching and referral public healthcare facility in East and Central Africa, with over 1,800 bed capacity [26]. In KNH, specimens submitted to the Microbiology laboratory for culture are routinely collected at the clinicians’ discretion, based on clinical suspicion of infection or as part of routine workup. In 2015, the laboratory processed a total of about 20,693 specimens as follows: 4731 blood cultures, 1614 skin and soft tissue, 5365 urine, 2489 stool, 2256 cerebrospinal fluid and 4238 others. The KNH Internal Medicine department comprises of 8 wards each with about 60 inpatient admissions at any given time. This department has previously recorded the highest proportion of *Staphylococcus aureus* isolates in the hospital [27]. Patients are admitted with a range of general medical conditions including community acquired infection and HIV-related complications.

### Study design

Herein we describe antimicrobial susceptibility patterns of *Staphylococcus aureus* isolates cultured from patients within the Internal Medicine department of KNH. Routine antimicrobial susceptibility data spanning 3 years’ duration (2014 to 2016) was largely collected from the 8 medical wards in a retrospective review and combined with a small prospective cross-sectional study in a hybrid research study design. The purpose of the prospective cross-sectional descriptive study was to enable the investigator to capture a snapshot of the patient demographics and relevant clinical correlates of AMR by reviewing data from inpatient files in the ward. Strict inclusion criteria for research data involved the first bacterial isolate of a given species per patient per analysis period, irrespective of body site, antimicrobial susceptibility profile, or other phenotypical characteristics. Any instances of incomplete data or mismatched information were excluded.

### Laboratory testing

All specimen processed in the KNH Microbiology laboratory were inoculated in sheep blood agar, chocolate blood agar and CLED (cysteine-, lactose-, and electrolyte-deficient) media as appropriate and aerobically incubated overnight at 37 °C. Cultures grown underwent phenotypic characterisation through routine bench identification methods including description of colony morphology (golden yellow colonies) on blood agar and biochemical testing methods such as gram stain and catalase tests, before further processing using VITEK-2 (bioMérieux). The VITEK 2 (bioMérieux) Gram Positive (GP) identification card was used to identify *Staphylococcus aureus* subspecies *aureus*. Antimicrobial susceptibility testing was performed using the automated VITEK-2 (bioMérieux) system, in conformation to the CLSI M100-S24 Performance Standards for Antimicrobial Susceptibility Testing; Twenty-Fourth Informational Supplement [28].

Antibiotics tested included oxacillin (30 μg cefoxitin), penicillin G (10 units), clindamycin (2 μg), erythromycin (15 μg), gentamicin (10 μg), tobramycin (10 μg), levofloxacin (5 μg), moxifloxacin (5 μg), linezolid (30 μg), mupirocin (10 μg), nitrofurantoin (300 μg), rifampicin (5 μg), tetracycline (30 μg), tigecycline (15 μg), trimethoprim/sulfamethoxazole (1.25/23.75 μg), teicoplanin (30 μg) and vancomycin (30 μg). A cut off ≥4 μg/ml for oxacillin testing and positive cefoxitin screening of *Staphylococcus aureus* isolates was reported as MRSA as a percentage of out of all *Staphylococcus aureus* isolates, as per the CLSI guidelines. Inducible clindamycin resistance testing was performed by VITEK 2 and together with its in-built automated Advanced Expert System (AES) was able to interpret test findings and adjust clindamycin susceptibility accordingly [29].

### Quality assurance

Quality control protocols were followed by the laboratory personnel, guided by specific internal standard operating procedures to enhance quality of specimen processing and storage, in efforts to minimise pre-analytical, analytical and post-analytical errors. Once received in the laboratory, careful scrutiny of the specimens was done, with rejection criteria applied to those which were deemed unfit for processing, such as mislabelled or contaminated specimen. After sorting, proper incubation and storage of specimens was ensured before processing, including refrigeration of certain specimens where appropriate. Standard ATCC (American Type Culture Collection) reference micro-organisms were used to check the performance of culture media. Sterility testing of media was done to ensure that there was no contamination of cultures. Verification of VITEK-2 results was done and inter-method comparison performed with offline manual methods such as Kirby-Bauer disk diffusion techniques. The laboratory has existing in-built controls and quarterly external quality checks (from specimen processing all through to VITEK reporting) through the World Health Organization – National Institute for Communicable Diseases, South Africa (WHO/NICD) and United Kingdom National External Quality Assurance Service (UK/NEQAS).

### Data analysis

Analysis of the results with special emphasis on non-duplicate *Staphylococcus aureus* isolates was done. The AST results were imported from the VITEK-2 system into the WHONET 5.6 software (World Health Organization) which was used to analyse the data, together with SPSS statistical software (Additional file 1). Only the first isolate of a given species per patient, irrespective of body site, per analysis period was selected by the analytical software in order to calculate the cumulative susceptibility percentage rates using CLSI breakpoints. This was cross-checked by the investigators to ensure conformity to the CLSI recommendations. All antimicrobial susceptibility data was stored in the KNH Microbiology electronic database.

## Results

### Isolate distribution

A total of 187 non-duplicate *Staphylococcus aureus* isolated over the three-year period were identified. About 34% (63/187) of the isolates were cultured in the year 2014, 38% (71/187) in 2015 and 28% (53/187) in 2016. The majority of isolates were cultured from skin and soft tissue (84%), followed by blood (10%) and urine (2%). Other isolates were obtained from pleural fluid, ascitic fluid, sputum and cerebrospinal fluid. Isolate distribution among the various clinical specimens has been shown in Table 1.

**Table 1:** Isolate distribution and their proportions among different specimen types

Year	2014	2015	2016	Overall (2014–2016)
Specimen type	n (%)	n (%)	n (%)	n (%)
Pus	58 (92%)	59 (83%)	40 (76%)	157 (84%)
Blood	4 (6%)	6 (9%)	8 (15%)	18 (10%)
Urine	–	3 (4%)	1 (2%)	4 (2%)
Miscellaneous^a^	1 (2%)	3 (4%)	4 (8%)	8 (4%)
TOTAL	63 (100%)	71 (100%)	53 (100%)	187 (100%)

^a^These include pleural fluid, ascitic fluid, sputum and cerebrospinal fluid

### Antimicrobial susceptibility of *Staphylococcus aureus* isolates

**Fig. 1:**
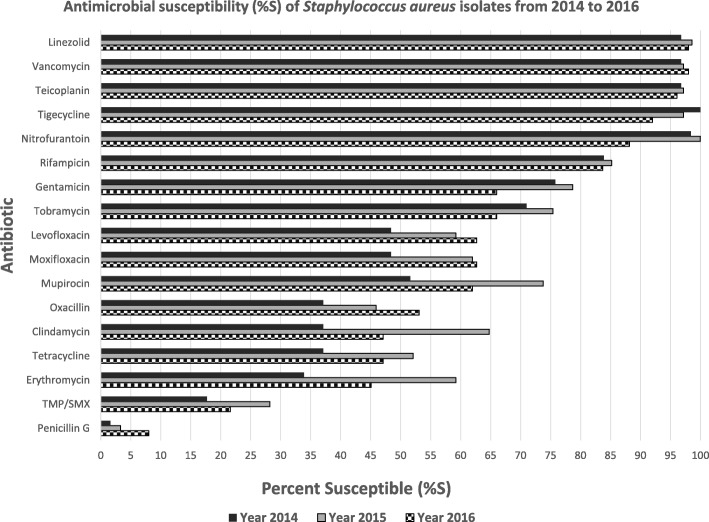
Antimicrobial susceptibility of *Staphylococcus aureus* isolates from 2014 to 2016

Antimicrobial susceptibility data for the *Staphylococcus aureus* isolates was reported on a yearly basis (as per CLSI guidelines for antibiogram reporting) in Fig. 1. Across the years 2014 up to 2016, *Staphylococcus aureus* demonstrated poor susceptibility to trimethoprim-sulfamethoxazole (17.7–28.2%) with moderate susceptibility to clindamycin, tetracycline and fluoroquinolones. Good susceptibility was seen to gentamicin and rifampicin and there was excellent susceptibility to linezolid, teicoplanin and vancomycin. Mupirocin susceptibility was less than 50% for the 3 years of study.

### Methicillin-resistant *Staphylococcus aureus*

**Fig. 2:**
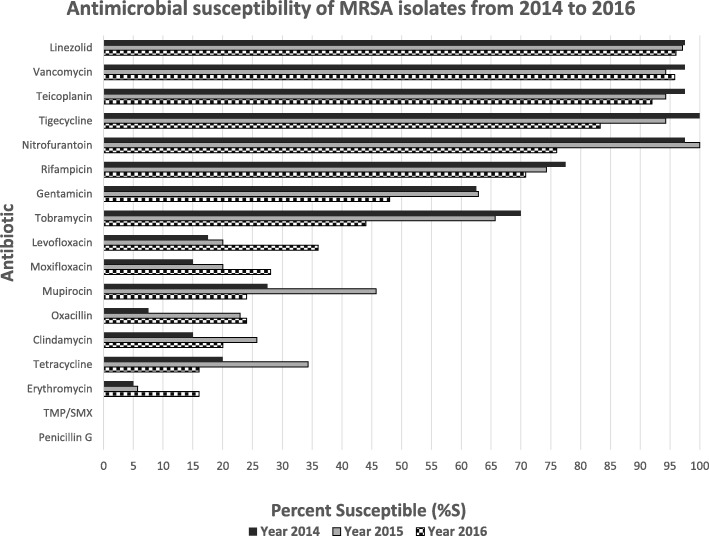
Antimicrobial susceptibility of Methicillin-resistant *Staphylococcus aureus* isolates from 2014 to 2016

Across the three years, there were 100 methicillin-resistant isolates, whereas specifically, MRSA was identified in 40 (40%) isolates in 2014, 35 (35%) in 2015 and 25 (25%) in 2016. Between 2014 and 2016 the overall MRSA prevalence was 53.4% (100/187 isolates). The majority of these isolates (80/100, 80%) were from skin and soft tissue infections, reflective of the overall distribution of *Staphylococcus aureus* isolates among different specimen types (as shown in Table 1). The antimicrobial susceptibility of the MRSA isolates is shown in Fig. 2. Excellent susceptibility was retained to linezolid, tigecycline, teicoplanin and vancomycin. In general, there was a declining trend in antibiotic susceptibility of these isolates across the years from 2014 to 2016, just like the methicillin susceptible counterparts. Isolates tended to demonstrate lower susceptibility per antibiotic in 2016 as compared to 2014, alluding to a yearly increase in antimicrobial resistance. This has been demonstrated graphically in comparative bar graphs (Figs. 1 and 2).^1^

### Patient demographics and clinical characteristics

In the small prospective descriptive cross-section, a total of 155 medical ward inpatients with positive bacterial cultures and AST results were indiscriminately sampled upon admission, capturing their demographic and clinical information. Sex distribution of these patients revealed 93 females (60%) and 62 males (40%). Their median age was about 48 years.

Out of these 155 patients with positive cultures, there were 17 non-duplicate *Staphylococcus aureus* isolates whereas the remaining 138 isolates represented other *Staphylococcus* species as well as other Gram positive and Gram negative bacteria. Cefoxitin screening revealed 59% MRSA prevalence from this data subset. The other clinical characteristics are as follows:

### Patient comorbidities

Majority of patients (109/155, 70%) had recognised comorbidities on admission. Out of the total patients, 49 (32%) had renal failure, 34 (22%) had diabetes mellitus, 27 (17%) were HIV seropositive, 24 (16%) had malignancy whereas 37 (24%) had other comorbidities.

### Empiric antibiotic therapy

Ninety-eight patients (63% of the total) had received empiric antibiotic therapy by the time a culture specimen was obtained, and these were grouped into the main antibiotic classes. Of these patients, 70 (70/98, 71%) had been empirically treated with a cephalosporin. Twenty-two patients (22/98, 22%) had been treated with a nitroimidazole such as Metronidazole. Twenty-one patients (21/98, 21%) had been treated with a penicillin whereas eight (8/98, 8%) had been treated with a carbapenem. Other antibiotics given empirically include macrolides (11/98, 11%), quinolones (13/98, 13%), aminoglycosides (4/98, 4%) and Vancomycin (4/98, 4%). Antibiotics scarcely prescribed included Linezolid (1/98, 1%). Overall, the median duration of empiric antibiotic therapy was 4 days prior to specimen collection for culture.

### Use of instrumentation and devices

Majority of patients (144/155, 93%) had an indwelling device or form of instrumentation. Most of them (138/155, 89%) had an intravenous line in situ. Other forms of instrumentation used included urinary catheters (49/155, 32%), nasogastric tubes (7/155, 5%), central venous catheters (3/155, 2%), haemodialysis catheters (21/155, 14%) among others (15/155, 10%).

### Duration of inpatient stay before specimen collection

The median duration of hospital stay before culture specimen collection was about 4 days. The minimum number of days spent in the ward before specimen collection was one day, whereas the longest admission period realised over the course of this study was 139 days.

## Discussion

MRSA prevalence is poorly reported in many African nations and according to the 2014 WHO report on antimicrobial resistance, Kenyan data was not recorded [2]. We observed 53.4% methicillin-resistance amongst significant *Staphylococcus aureus* isolates in the adult general medical population of KNH. This was comparable to 50.6% MRSA observed amongst paediatric surgical patients in 2014 [30], and 46.5% MRSA rate reported with the *mecA* resistance gene in *Staphylococcus aureus* from paediatric ICU in KNH [31]. In our study, the major source of MRSA infection was isolated from skin and soft tissue (80%). This is comparable to other regional figures, such as Eritrea which recorded 71.9% MRSA from pus specimens [7]. The higher frequency of MRSA in pus samples as compared to blood and other specimen has been reported, especially in diabetic foot infections, surgical wounds, and burn patients [7, 32–35].

Local prevalence rates of MRSA have been increasing in KNH since 2003 when a rate of 27.7% was reported [36]. Molecular gene typing of MRSA locally in Kenyan public and private facilities has demonstrated significant presence of epidemic clones [37]. There is a sharp contrast between methicillin resistance reported in public hospitals such as KNH versus other private hospitals in Nairobi [38]. In 2013, three local public health facilities reported 84.1% MRSA prevalence accompanied by *mecA* gene typing [39]. On the other hand, 2 private hospitals maintained low prevalence of about 3.7% during 2011–2013 and about 6.5% in 2014 using the automated identification system VITEK-2 (bioMérieux) [38, 40, 41].

KNH introduced the automated VITEK-2 system in 2013, as it confers the advantage of greater accuracy, reliability and speed of isolate identification and antimicrobial susceptibility testing [42] than conventional manual methods. VITEK-2 (bioMérieux) accuracy has been widely reported in literature showing between 95 and 99% correct *Staphylococcus aureus* species identification [43, 44], 98.3% categorical agreement for staphylococcus testing [45] and negligible rates of false positives as low as 1.1% [46]. This greater accuracy in identification of *Staphylococcus aureus* and other gram-positive cocci has continually been independently validated ever since the redesign of the VITEK 2 g-positive (GP) identification card [47].

Reasons for differences in MRSA rates between public and private hospitals are likely to be multifactorial. There are marked sociodemographic differences between patient population, antibiotic exposure, differences in the hospital environment as well as in infection prevention and control (IPC) practices. It is noteworthy that for one of these private hospitals with effective IPC protocols, nasal carriage of MRSA among healthcare workers was reported to be 0% [48] contrasting with 18.9% MRSA carriage amongst 180 KNH healthcare workers [49]. Ugandan studies have noted MRSA carriage rates of up to 8% among patients [50] and overall carriage rate of 13% among health workers [51]. High rates of MRSA carriage amongst health care workers gives particular cause for concern given poor infection prevention and control measures in resource poor settings. A 2013 literature review assessing burden of MRSA in Africa suggested socioeconomic conditions, communicable and non-communicable diseases and selection pressure due to antibiotic overutilization as factors influencing variable MRSA prevalence in the different localities [8]. Of note, KNH is a tertiary referral public hospital which receives patients of low to middle income status directly from the community as well as referrals from other public primary and secondary healthcare facilities with a higher burden of comorbidities influencing MRSA prevalence such as malignancy, Human Immunodeficiency Virus (HIV) infection and tuberculosis [8]. In particular, HIV has been described as a driver to evolution of antimicrobial resistance in *Staphylococcus aureus* [52]. Apart from HIV infection, our study highlighted other key comorbidities such as malignancy, diabetes mellitus and renal failure among inpatients. In addition, other Kenyan studies have corroborated the tendency for MRSA infections to be isolated at public healthcare facilities (such as KNH) which serves as a major referral centre for the economically disadvantaged living in urban informal settlements [39]. All these reasons, together with high antibiotic consumption in our public healthcare facility, can easily translate to higher burdens of antimicrobial resistance.

Traditionally, antibiotic overuse has been described as a major driver of antimicrobial resistance and availability of antibiotics has been noted to account for regional differences in AMR rates [8]. We noted in our study that there was high susceptibility to antibiotics rarely prescribed, such as Linezolid. On the other hand, high rates of resistance were noted in the case of antibiotics such as mupirocin and trimethoprim/sulfamethoxazole which in Kenya are obtainable over-the-counter for treatment of various infections. Mupirocin resistance attributable to overconsumption has been demonstrated among patients in various countries globally, with rates as high as 65% in some settings [53–56]. High resistance to trimethoprim-sulfamethoxazole has been demonstrated in many African countries [57–59] and may possibly point towards overexposure in our local setting where it is largely used for HIV prophylaxis, as confirmed in other studies [40, 59]. Furthermore, the widespread trimethoprim resistance mediated by the dfrG gene among African patients and imported from ill-returning travellers to Europe pose a grave concern, foreshadowing the impotence of this drug for empirical use in treating *Staphylococcus aureus* infections [60]. Similarly as in our study, resistance to tetracycline and erythromycin has been noted in other African patients with import into the European continent, sometimes resulting in fatal epidemic nosocomial outbreaks [61–63].

Inter-laboratory variability in isolate identification and testing can influence MRSA reporting, with molecular methods demonstrating lower rates than phenotypic ones [8]. For example, phenotypic misidentification of coagulase-negative staphylococcus (CoNS) as *Staphylococcus aureus* can pose as possible confounders contributing to overestimation of methicillin resistance [38]. CoNS are largely methicillin-resistant commensals found on anterior nares, skin and mucous membranes. Often they cohabitate with *Staphylococcus aureus*, and may result in frequent isolation together from the same specimen collected [64]. Instances of misidentification of *Staphylococcus aureus* has also been cited with use of chromogenic agar plates [65] and molecular polymerase chain reaction (PCR) methods [64]. Methicillin resistance *mecA* gene typing is present on CoNS and *Staphylococcus aureus*, and thus it is not uncommon to have false positives of MRSA reported [66]. A possible solution to this involves concurrent detection of *Staphylococcus aureus*-specific gene markers such as *nuc* [64] and *orfX* [66]. Implementation of whole genome sequencing and bioinformatics aids in laboratory testing and reporting of significant isolates of concern. Inasmuch as these genetic assays improve accuracy, they are expensive and scarcely available in African countries. Cheaper methods involve the use of laboratory bench phenotypic methods such as coagulase testing among other sequel biochemical techniques such as mannitol salt agar and deoxyribonuclease (DNase) [67] to augment the VITEK-2 identification methods. Ultimately, it has been suggested that the background local or regional MRSA prevalence should always be taken into account during reporting [64].

Study limitations include over-reliance on VITEK-2 for species identification in preference to manual phenotypic methods, as a result of embracing automated laboratory methods in efforts to benchmark with international practice. This may be true for other hospitals in Africa that may be following similar trends [38]. The retrospective review was also prone to design-related limitations such as missing clinical information including patient demographics and clinical surrogates such as antibiotics prescribed, use of instrumentation devices, patient comorbidities, duration of inpatient stay, morbidity and mortality outcomes. We attempted to give a snapshot of the clinical scenarios by undertaking a small prospective descriptive study, albeit limited by available resources. The principal investigators were also unable to carry out novel molecular techniques and implement bioinformatics due to financial limitations. Lastly, this study was not designed to stratify between hospital-acquired and community-acquired infections.

Ultimately, the controversy on true versus false MRSA identification and reporting can only be clearly settled by a combination of manual phenotypic methods on the laboratory bench and multiple gene sequencing, which is very expensive and widely unavailable, especially in developing countries. Building the capacity of local microbiology laboratories to embrace bioinformatics and molecular methods in resistance testing would be ideal, and is highly recommended to improve diagnostic accuracy and overcome discrepancies arising from inter-laboratory differences. Some noteworthy efforts towards molecular characterisation of resistant *Staphylococcus aureus* have already begun in some local private healthcare facilities, notwithstanding financial challenges [37]. Ultimately, there is a need for standard external reference laboratories in our region which can perform molecular testing and surveillance of such critical isolates from various local laboratories, eventually contributing towards a central national database of resistance data.

## Conclusion

Careful interrogation of antimicrobial susceptibility results is important to uproot any red herrings and reserve genuine cause for alarm, as this has a critical bearing on health and economic outcomes for a population. Inter-laboratory and interpersonal differences that may exist between different facilities underscore the need for better clinician and laboratory interfacing, as part of antimicrobial stewardship efforts. Embracing new technology and integration of molecular methods in clinical practice is paramount towards dispelling disparities and disseminating clear accurate information to guide clinical decision-making regarding resistance data of high concern. This informs key policy-makers on effective strategies to fight the problem as well as efficient allocation of scarce healthcare resources. Otherwise without such multi-disciplinary collaborative efforts, one would wonder– are we overestimating or underestimating antimicrobial resistance trends in the developing world, and what are the clinical and health economic implications thereof? This baseline study unearthed as many questions as answers, with important lessons learnt for clinicians, microbiologists, hospital administrators and healthcare facilities in Africa, and in the world at large that is grappling with the impending post-antibiotic era.

## Additional file

Additional file 1: MRSA study, Supplementary Material. (SAV 101 kb)

## Data Availability

All data generated or analysed during this study are included in this published article [and its supplementary information files].

## Abbreviations

%S: Percent Susceptible
AST: Antimicrobial Susceptibility Testing
CLSI: Clinical & Laboratory Standards Institute
CoNs: Coagulase-negative *Staphylococcus aureus*
HIV: Human Immunodeficiency Virus
ICU: Intensive Care Unit
KNH: Kenyatta National Hospital
MRSA: Methicillin-resistant *Staphylococcus aureus*
TMP/SMX: Trimethoprim/Sulfamethoxazole
WHO: World Health Organization

## Footnotes

1 The percent susceptible for each organism/antibiotic combination was generated by including the first isolate of that organism encountered on a given patient. Clindamycin susceptibilities were adjusted for inducible clindamycin resistance. Nitrofurantoin activity is suggested for urine isolates.
